# Numerical analysis of spray characterization of blends of hydrous ethanol with diesel and biodiesel

**DOI:** 10.1038/s41598-024-56444-0

**Published:** 2024-03-08

**Authors:** Vardelli Shanthan, Jiwak Suryawanshi, Rahul Tarodiya, Akshay Loyte, Yuvarajan Devarajan, Nandagopal Kaliappan

**Affiliations:** 1 Department of Mechanical Engineering, Visvesvaraya National Institute of Technology, Nagpur, Maharashtra India; 2 Department of Mechanical Engineering, Saveetha School of Engineering, SIMATS, Saveetha University, Chennai, Tamil Nadu India; 3 Department of Mechanical Engineering, Haramaya Institute of Technology, Haramaya University, Dire Dawa, Ethiopia

**Keywords:** Penetration length, Biodiesel, Ethanol, Nozzle diameter, Injection pressure, Spray, Engineering, Mechanical engineering

## Abstract

The spray characteristics of a fuel greatly influence the combustion as it affects the formation of an air–fuel mixture, which directly impacts the performance and emissions of the engine. This study investigates the physical injection spray characteristics of biofuels to optimize the engine operating parameters for their effective utilization. For the analysis of the spray characteristics of pure diesel (D100), 80% diesel—20% biodiesel (D80B20), 80% diesel—10% biodiesel—10% pure ethanol (D80B10E10), and 80% diesel—10% biodiesel—10% hydrous ethanol (D80B10HE10) are investigated. Computational Fluid Dynamics (CFD) modeling of a constant volume chamber under non-evaporative conditions is performed to conduct numerical analysis. The chamber pressure of 2 and 2.5 MPa and nozzle injection diameter of 0.126 mm, 0.15 mm, and 0.2 mm are considered to conduct the simulations. The variation in spray penetration length is analyzed and discussed for the injection of different fuel blends at different initial conditions. It is observed from numerical results that the high-density fuel blend D80B20 has a penetration length of 10.695% and 15.805% higher than pure diesel and D80B10HE10 blends, respectively. For pure diesel, with an increase in nozzle diameter from 0.126 mm to 0.15 mm and 0.2 mm, the penetration length is increased by 20% and 32%, respectively, and with an increase in pressure from 2 MPa to 2.5 MPa, penetration length is decreased by 14.62%. From this study, it can be concluded that biofuels like biodiesel and hydrous ethanol can be used with diesel in blended form over pure ethanol. Compared to pure ethanol, hydrous ethanol gives cost benefits and better spray characteristics.

## Introduction

The rapid growth in the automobile industry leads to consuming huge amounts of fossil fuels. With the consumption of fossil fuels at a higher rate, most of the world's oil supplies are likely to be drained within the next few decades. Further, the use of fossil fuels has some significant environmental concerns, including air pollution, water pollution, and climate change. Thus, in recent years, the suitability of an alternate fuel, which can replace the use of some amount of fossil fuel and reduce the emissions produced from them, is being explored^1,2^.

Biofuels such as biodiesel and bioethanol produced from organic matter such as plants, algae, and organic waste are commonly accepted as alternate fuels. In recent years, the use of these alternate fuels has become more common in the field of Internal Combustion (IC) engines^3–5^. These fuels have gained importance as an alternate fuel because they are renewable fuels, non-toxic, and produce low emissions from the engine. They can also be used with conventional diesel fuel in the form of fuel blends to reduce the quantity of diesel used and emissions. The properties of these blends are near to the conventional diesel fuel, and only minor or no changes are required in the engine to use these fuel blends^4–7^.

Biodiesel is produced by the process of transesterification from animal fat and vegetable oils. Transesterification is the reaction where oil/fat reacts with alcohol in the presence of a catalyst to form an alkyl ester and byproduct as glycerol. For producing biodiesel, the reaction converts one mole of oil/triglycerides with three moles of alcohol to three moles of alkyl ester and one mole of glycerol^8–10^. Equation (1) shows the reaction of transesterification. Biodiesel can be produced from edible oils of soyabean, palm, jatropha etc. This motivates farmers to grow crops specifically for this purpose which leads to significant rise in food prices as there is limited agricultural land and water resources available^11–13^. So, the waste cooking oil (WCO) replaced the use of edible oils in biodiesel production. Biodiesel produced from WCO through homogeneous alkaline transesterification using NaOH as the catalyst, and the oil-to-alcohol at a ratio of 6:1 M in a batch reactor^14,15^.

1 $$\begin{array}{*{20}c} {{\varvec{H}}_{{\varvec{2}}} \user2{C - OCOR^{\prime}}} & {} & {} & {} & {\user2{ROCOR^{\prime}}} & {} & {{\varvec{H}}_{{\varvec{2}}} \user2{C - OH}} \\ {{\varvec{H}}\mathop {\varvec{C}}\limits_{\user2{|}}^{\user2{|}} \user2{ - OCOR^{\prime\prime}}} & \user2{ + } & {{\varvec{3ROH}}} & {\overset {{\varvec{Catalyst}}} \leftrightarrows } & {{\varvec{RO}}\mathop {\varvec{C}}\limits_{\user2{ + }}^{\user2{ + }} \user2{OR^{\prime\prime}}} & \user2{ + } & {{\varvec{H}}\mathop {\varvec{C}}\limits_{\user2{|}}^{\user2{|}} \user2{ - OH}} \\ {{\varvec{H}}_{{\varvec{2}}} \user2{C - OCOR^{\prime\prime\prime}}} & {} & {} & {} & {\user2{ROCOR^{\prime\prime\prime}}} & {} & {{\varvec{H}}_{{\varvec{2}}} \user2{C - OH}} \\ {{\varvec{Triglyceride}}} & {} & {{\varvec{Alcohol}}} & {} & {{\varvec{Alkylester}}} & {} & {{\varvec{Glycerol}}} \\ \end{array}$$

Ethanol is another alternative fuel, which is a renewable fuel used over the last two decades to substitute fossil fuels and reduce emissions partly. It is produced by fermenting the sugar and starch components of crops like corn, sugarcane, and wheat, and it can also be produced from cellulosic biomass such as wood and agricultural residues^16,17^. Ethanol is preferred over other alcohols as it has an oxygen content of up to 35%^18^. In the production process of ethanol, after fermentation, dehydration is done to remove water up to azeotropic conditions (hydrous ethanol). From that condition, the production of anhydrous ethanol is relatively expensive in terms of cost and energy consumption for distillation process^19,20^. It was reported that energy consumption constitutes as much as 37% of the total energy input with only 6% net energy gain attributed to the fuel^21,22^. So, to conserve energy and reduce costs, hydrous ethanol is used. The use of hydrous ethanol in diesel engines proves advantageous contributing to the reduction of exhaust emissions. Water content in hydrous ethanol leads to phase separation, which can be eliminated by adding biodiesel or any higher alcohol^23,24^.

The fuel spray characteristics and formation of the air–fuel mixture affect the combustion of the diesel engine, which has a huge impact on emissions produced in the engine. Fuel spray characteristics include spray tip penetration (penetration length) and spray cone angle. Penetration length is defined as the distance between the nozzle exit and to tip of the spray. The spray cone angle is defined as the angle between the lines tangent to spray lateral edges^25^. Fuel spray analysis can be experimentally and numerically by using CFD codes. The effect of injection pressure and various ambient conditions on fuel spray in a constant volume chamber was experimentally investigated by Lee et al.^26^. They reported that there is a change in penetration length due to the density difference between diesel and gasoline. Corral-Gómez et al.^25^ conducted a similar kind of experimental study as Lee et al.^26^. They reported that blends with higher density show increased penetration length, and those having less kinematic viscosity show lesser penetration length. Agarwal et al.^27^ experimentally investigated the effect of ambient pressure on spray characteristics in a constant volume chamber using Karanja biodiesel blends. They reported that ambient pressure directly affects the spray pattern. As the chamber pressure increases, penetration length decreases. Similar results are also reported by^28–31^. Algayyim et al.^32^ investigated the impact of injector hole diameter on spray behavior. They observed that by increasing the injector hole diameter, the penetration length of the spray increases.

Kuti et al.^33^ numerically analyze the spray characteristics of waste cooking oil (WCO) biodiesel and diesel using CFD code Converge. They reported that the WCO biodiesel has having higher penetration length than diesel. Similar results are also reported by^34,35^. Haq et al.^36^ used CFD code ANSYS FLUENT to investigate the spray behavior of castor and jatropha biodiesel. They reported the higher penetration length and narrower cone angle of biodiesel compared to diesel. Park et al.^37^ conducted experimental spray analysis with varying fuel and ambient temperatures. They reported that the increase in fuel temperature decreases spray tip penetration.

From the literature review, it is found that many studies are being conducted on the spray characteristics of diesel and biodiesel by using both experimental and numerical methods. However, the fuel spray characteristics of blends of alternate fuels have not been much explored, as conventional fuels are being substituted partly or completely by alternate fuels. In this study, various fuel blends of diesel, WCO biodiesel, and hydrous ethanol are used to study the spray characteristics. The analysis is conducted under non-evaporating conditions with varying ambient pressures and nozzle injection diameters. Abbreviations and percentages of different fuel blends (in volume percentage) that are used to investigate the spray characteristics are presented in Table 1.

**Table 1:** Fuel blend ratios were used for the simulation study.

Fuel blends	Diesel (D)	Waste cooking oil biodiesel (B)	Ethanol (E)	Hydrous ethanol
D100	100 (vol %)	–	–	–
D80B20	80 (vol %)	20 (vol %)	–	–
D80B10E10	80 (vol %)	10 (vol %)	10 (vol %)	–
D80B10HE10	80(vol %)	10 (vol %)	–	10 (vol %)

## Methodology

This study comply with relevant institutional, national, and international guidelines and legislation. The multiphase modeling for the spray formation is performed by adopting the Eulerian–Lagrangian approach. CFD code ANSYS FLUENT is used to conduct the simulations. The gas phase is treated as a continuous phase, and the fuel droplets are modeled as a dispersed phase. The governing equations for continuity, momentum, and energy are given under.

Continuity equation:

2 $$\frac{{\partial {\varvec{\rho}}}}{{\partial {\varvec{t}}}} + \nabla .(\user2{\rho V}) = 0$$

The conservation of momentum equation:

3 $$\frac{\partial }{{\partial {\varvec{t}}}}\user2{(\rho V) + }\nabla \user2{.(\rho V V)} = - \frac{{\partial {\varvec{p}}}}{{\partial {\varvec{x}}}}\user2{ + }\nabla \user2{.}\overline{\overline{\tau }} \user2{ + \rho g + S}_{{\varvec{M}}}$$

The conservation of energy equation:

4 $$\frac{\partial }{{\partial {\varvec{t}}}}\user2{(\rho e) + }\nabla \user2{.(\rho eV) = - p}\nabla \user2{V + }\nabla \user2{(k}\nabla \user2{T) + }\phi$$

where ρ is the density of fuel, V is the instantaneous velocity vector, p is the pressure, $\nabla .\overline{\overline{\tau }}$ is the stress tensor, ρg is the gravitational force, e is internal energy, k is the thermal conductivity, ϕ is the dissipation function, and S_M_ is the source term which the ratio of drag force (F_D_) to the volume of the cell.

The two-way coupled approach is adopted to solve the governing equations for both phases, and the exchange of mass, momentum, and energy between the dispersed and continuous phase is calculated as^38^

Momentum Exchange:

5 $${\varvec{F}}_{D} \user2{ = }\sum {\left( {\frac{{\user2{18\mu C}_{{\varvec{d}}} {\varvec{Re}}}}{{{\varvec{\rho}}_{{\varvec{p}}} {\varvec{d}}_{{\varvec{p}}}^{{\varvec{2}}} {\varvec{24}}}}\left( {{\varvec{V}}_{{\varvec{p}}} \user2{ - V}} \right)} \right)} \dot{\user2{m}}_{{\varvec{p}}} \user2{\Delta t}$$

where μ is the viscosity of the fluid, ρ_p_, d_p,_ and V_p_ is the density, diameter, and velocity of the particle, Re is the Reynolds number, V is the velocity of the fluid, C_d_ is the drag coefficient, $\dot{\user2{m}}_{{\varvec{p}}}$ is the mass flow rate of the particles and Δt is the time step.

Mass Exchange:

6 $$\user2{M = }\frac{{\user2{\Delta m}_{{\varvec{p}}} }}{{{\varvec{m}}_{{\user2{p,0}}} }}\dot{\user2{m}}_{{\user2{p,0}}}$$

where m_p_ is the mass of the particle and $\dot{m}_{p,0}$ is the initial mass flow rate of injection.

Heat Exchange:

7 $$\user2{Q = }\frac{{\dot{\user2{m}}_{{\user2{p,0}}} }}{{{\varvec{m}}_{{\user2{p,0}}} }}\left[ {\left( {{\varvec{m}}_{{{\varvec{p}}_{{{\varvec{in}}}} }} \user2{ - m}_{{{\varvec{p}}_{{{\varvec{out}}}} }} } \right)\left( {\user2{ - H}_{{{\varvec{lat}}_{{{\varvec{ref}}}} }} \user2{ + H}_{{{\varvec{pyrol}}}} } \right)\user2{ - m}_{{{\varvec{p}}_{{{\varvec{out}}}} }} \int\limits_{{{\varvec{T}}_{{{\varvec{ref}}}} }}^{{{\varvec{T}}_{{{\varvec{p}}_{{{\varvec{out}}}} }} }} {{\varvec{c}}_{{{\varvec{p}}_{{\varvec{p}}} }} {\varvec{dT}}} \user2{ + m}_{{{\varvec{p}}_{{{\varvec{in}}}} }} \int\limits_{{{\varvec{T}}_{{{\varvec{ref}}}} }}^{{{\varvec{T}}_{{{\varvec{p}}_{{{\varvec{in}}}} }} }} {{\varvec{c}}_{{{\varvec{p}}_{{\varvec{p}}} }} {\varvec{dT}}} } \right]$$

where $\dot{m}_{p,0}$ is the initial mass flow rate of injection, m_p,0_ is the initial mass of the particle, $m_{{p_{in} }}$ and $m_{{p_{out} }}$ is the mass of the particle on cell entry and exit, ${\text{c}}_{{{\text{p}}_{{\text{p}}} }}$ is heat capacity of the particle, H_pyrol_ heat of pyrolysis, ${\text{T}}_{{{\text{p}}_{{{\text{in}}}} }}$ and ${\text{T}}_{{{\text{p}}_{{{\text{out}}}} }}$ is the temperature of a particle at cell entry and exit, T_ref_ is the reference temperature for enthalpy and $H_{{lat_{ref} }}$ is latent heat at reference condition.

To compute the droplet drag coefficient, the droplets are assumed to be spherical throughout the flow field. The drag coefficient of the spherical droplet is calculated as^39^

8 $${\varvec{C}}_{{\user2{d,sphere}}} \user2{ = }\left\{ {\begin{array}{*{20}c} {\frac{{{\varvec{24}}}}{{{\varvec{Re}}}}\user2{(1 + }\frac{{\varvec{1}}}{{\varvec{6}}}{\varvec{Re}}^{{{{\varvec{2}} \mathord{\left/ {\vphantom {{\varvec{2}} {\varvec{3}}}} \right. \kern-0pt} {\varvec{3}}}}} \user2{)}} & {\user2{Re < 1000}} \\ {{\varvec{0}}\user2{.424}} & {\user2{Re > 1000}} \\ \end{array} } \right.$$

Further, a dynamic drag model is adopted to account for the droplet distortion effects, which linearly varies the drag of the sphere and disk. The drag coefficient of the droplet is calculated as^39^

9 $${\varvec{C}}_{{\varvec{d}}} \user2{ = C}_{{\user2{d,sphere}}} \left( {\user2{1 + 2}\user2{.632y}} \right)$$

where y is the droplet distortion, calculated as^39^

10 $$\frac{{d^{2} y}}{{dt^{2} }}\user2{ = }\frac{{{\varvec{C}}_{{\varvec{F}}} {\varvec{\rho}}_{{\varvec{2}}} {\varvec{W}}^{{\varvec{2}}} }}{{{\varvec{C}}_{{\varvec{b}}} {\varvec{\rho}}_{{\varvec{1}}} {\varvec{a}}^{{\varvec{2}}} }}\user2{ - }\frac{{{\varvec{C}}_{{\varvec{K}}} {\varvec{\sigma}}}}{{{\varvec{\rho}}_{{\varvec{1}}} {\varvec{a}}^{{\varvec{3}}} }}\user2{y - }\frac{{{\varvec{C}}_{{\varvec{d}}} {\varvec{\mu}}_{{\varvec{1}}} }}{{{\varvec{\rho}}_{{\varvec{1}}} {\varvec{a}}^{{\varvec{2}}} }}\frac{dy}{{dt}}$$

where C_F_, C_b_, C_K,_ and C_d_ are constants^38^.

In ANSYS Fluent, the simulation of the pressure-swirl atomizer involves the application of the Linearized Instability Sheet Atomization (LISA) model, which was developed by Schmidt et al.^40^. The LISA model encompasses processes such as film formation, sheet breakup, and atomization^41,42^. The breakup length is estimated as

11 $${\text{L}}_{{\text{b}}} { = }\frac{{\text{U}}}{{\Omega }}{\text{ln(}}\frac{{{\upeta }_{{\text{b}}} }}{{{\upeta }_{{0}} }}{)}$$

12 $${\text{U = C}}_{{\text{d}}} \sqrt {\frac{{2\Delta {\text{p}}}}{{\rho_{{\text{l}}} }}}$$

where U is total velocity, Ω is the maximum growth rate, Δp is pressure drop across injector and ln $\left(\frac{{\eta }_{b}}{{\eta }_{0}}\right)$ is an empirical sheet constant with a value of 12 obtained theoretically^38^.

To account for the instability, the primary and secondary breakup of two fluids based on the density differences Kelvin–Helmholtz (KH) and Rayleigh–Taylor (RT) breakup theory is adopted. Both methods are commonly used to simulate the droplet breakup. This is done by monitoring wave growth on the droplet's surface, with breakup occurring as a result of the instability that is growing the fastest under specific local conditions. Based on the KH model, the frequency of fastest fastest-growing wave and corresponding wavelength are expressed as^43,44^

13 $${\varvec{\varOmega}}_{{{\varvec{KH}}}} \user2{ = }\frac{{{\varvec{0}}\user2{.34 + 0}\user2{.38We}_{{\varvec{2}}}^{{{\varvec{1}}\user2{.5}}} }}{{\user2{(1 + Z)(1 + 1}\user2{.4T}^{{{\varvec{0}}\user2{.6}}} \user2{)}}}\sqrt {\frac{{\varvec{\sigma}}}{{{\varvec{\rho}}_{{\varvec{1}}} {\varvec{a}}^{{\varvec{3}}} }}}$$

14 $${\varvec{\varLambda}}_{{{\varvec{KH}}}} \user2{ = }\frac{{{\varvec{9}}\user2{.02a(1 + 0}\user2{.45Z}^{{{\varvec{0}}\user2{.5}}} \user2{)(1 + 0}\user2{.4T}^{{{\varvec{0}}\user2{.7}}} \user2{)}}}{{\user2{(1 + 0}\user2{.87We}_{{\varvec{2}}}^{{{\varvec{1}}\user2{.67}}} \user2{)}^{{{\varvec{0}}\user2{.6}}} }}$$

where We_2_ is the Weber number of gas, ρ_1_ is liquid density, σ is surface tension, a is the radius of the parent liquid drop and Ohnesorge number $\user2{Z = }{{{\varvec{We}}_{{\varvec{1}}}^{{{\varvec{0}}\user2{.5}}} } \mathord{\left/ {\vphantom {{{\varvec{We}}_{{\varvec{1}}}^{{{\varvec{0}}\user2{.5}}} } {{\varvec{Re}}_{{\varvec{1}}} }}} \right. \kern-0pt} {{\varvec{Re}}_{{\varvec{1}}} }}$, Taylor number $\user2{T = Z We}_{{\varvec{2}}}^{{{\varvec{0}}\user2{.5}}}$ and We_1_ is the Weber number of liquid, and Re is Reynolds number. The breakup of droplet parcels of radius a to form new droplets of radius r_c_ is such that.

15 $${\varvec{r}}_{{\varvec{c}}} \user2{ = B}_{{\varvec{0}}}{\varvec{\varLambda}}_{{{\varvec{KH}}}}$$

where B_0_ is a constant equal to 0.61^43^. During breakup, the parent particle diameter reduces due to a change in mass. The rate of change of radius is

16 $$\frac{{{\varvec{dr}}}}{{{\varvec{dt}}}}\user2{ = }\frac{{\user2{a - r}_{{\varvec{c}}} }}{{{\varvec{\tau}}_{{{\varvec{KH}}}} }}$$

where τ_KH_ is breakup time and given as

17 $${\varvec{\tau}}_{{{\varvec{KH}}}} = \frac{{{\varvec{3}}\user2{.726B}_{{\varvec{1}}} {\varvec{a}}}}{{\Omega_{KH} \Lambda_{KH} }}$$

where B_1_ is constant and its value lies between 10 and 60. KH model predicts the primary breakup of the fuel spray. RT model, along with the KH model, is used to predict the secondary breakup of droplets. The frequency of the fastest growing wave and corresponding wave number for the RT model is given by^44^

18 $${\varvec{\varOmega}}_{{{\varvec{RT}}}} \user2{ = }\sqrt {\frac{{{\varvec{2}}\left[ {\user2{ - g}_{{\varvec{t}}} \user2{(\rho }_{{\varvec{f}}} \user2{ - \rho }_{{\varvec{a}}} \user2{)}} \right]^{{{{\varvec{3}} \mathord{\left/ {\vphantom {{\varvec{3}} {\varvec{2}}}} \right. \kern-0pt} {\varvec{2}}}}} }}{{{\varvec{3}}\sqrt {\user2{3\sigma }} \user2{(\rho }_{{\varvec{f}}} \user2{ + \rho }_{{\varvec{a}}} \user2{)}}}}$$

19 $${\varvec{K}}_{{{\varvec{RT}}}} \user2{ = }\sqrt {\frac{{\user2{ - g}_{{\varvec{t}}} \user2{(\rho }_{{\varvec{f}}} \user2{ - \rho }_{{\varvec{a}}} \user2{)}}}{{\user2{3\sigma }}}}$$

where g_t_ is the droplet acceleration in the direction of droplet travel. After the breakup, the radius of newly formed droplets and breakup time is given by

20 $${\varvec{r}}_{{\varvec{c}}} \user2{ = }\frac{{\user2{\pi C}_{{{\varvec{RT}}}} }}{{{\varvec{K}}_{{{\varvec{RT}}}} }}\& {\varvec{\tau}}_{{_{{{\varvec{RT}}}} }} \user2{ = }\frac{{{\varvec{C}}_{{\varvec{\tau}}} }}{{{\varvec{\varOmega}}_{{{\varvec{RT}}}} }}$$

where C_RT_ and C_τ_ are constants of values 0.1 and 1^44^. Figure 1 shows the flow chart of simulation process.

**Figure 1:**
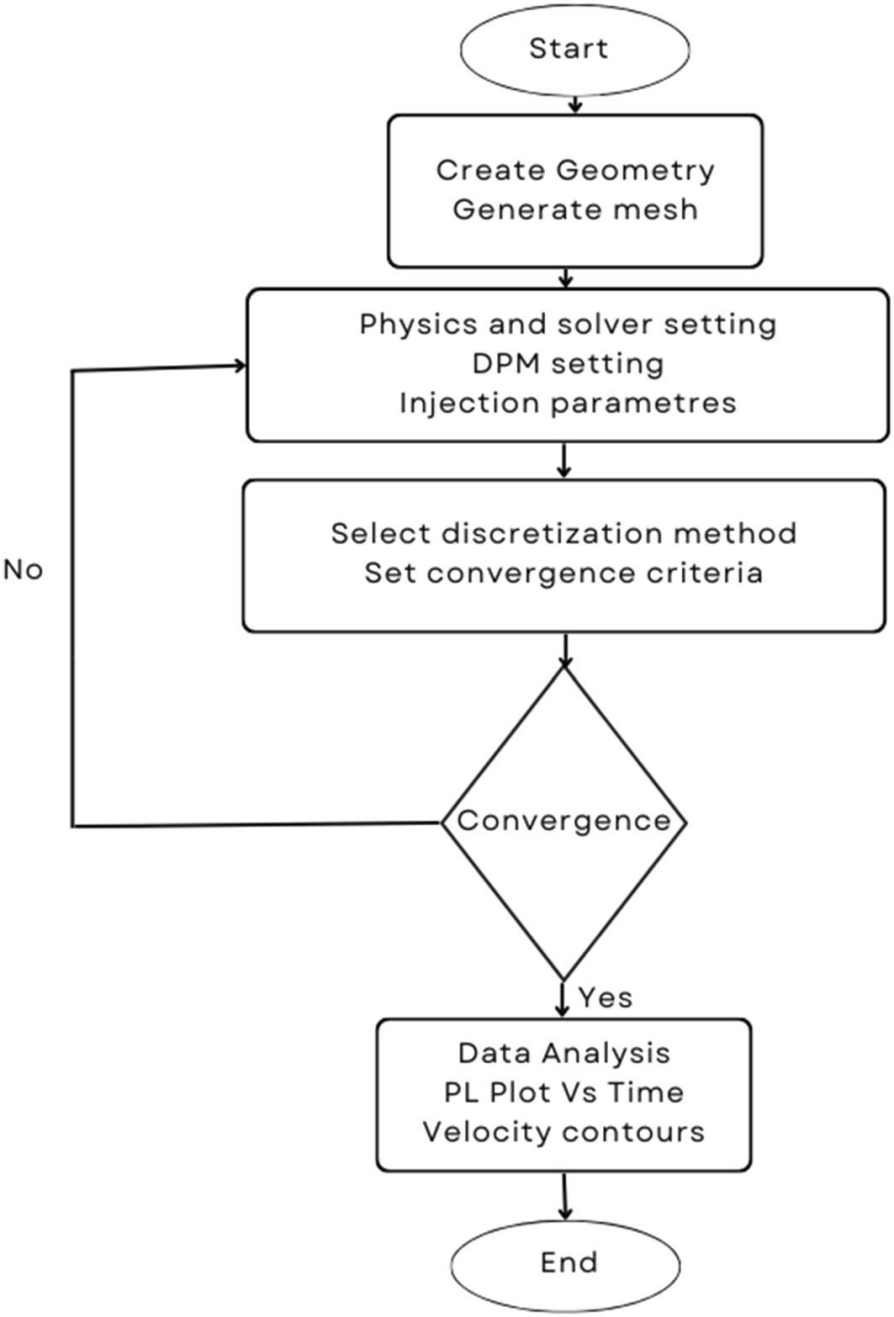
Flow chart of simulation process.

## Numerical setup

**Figure 2:**
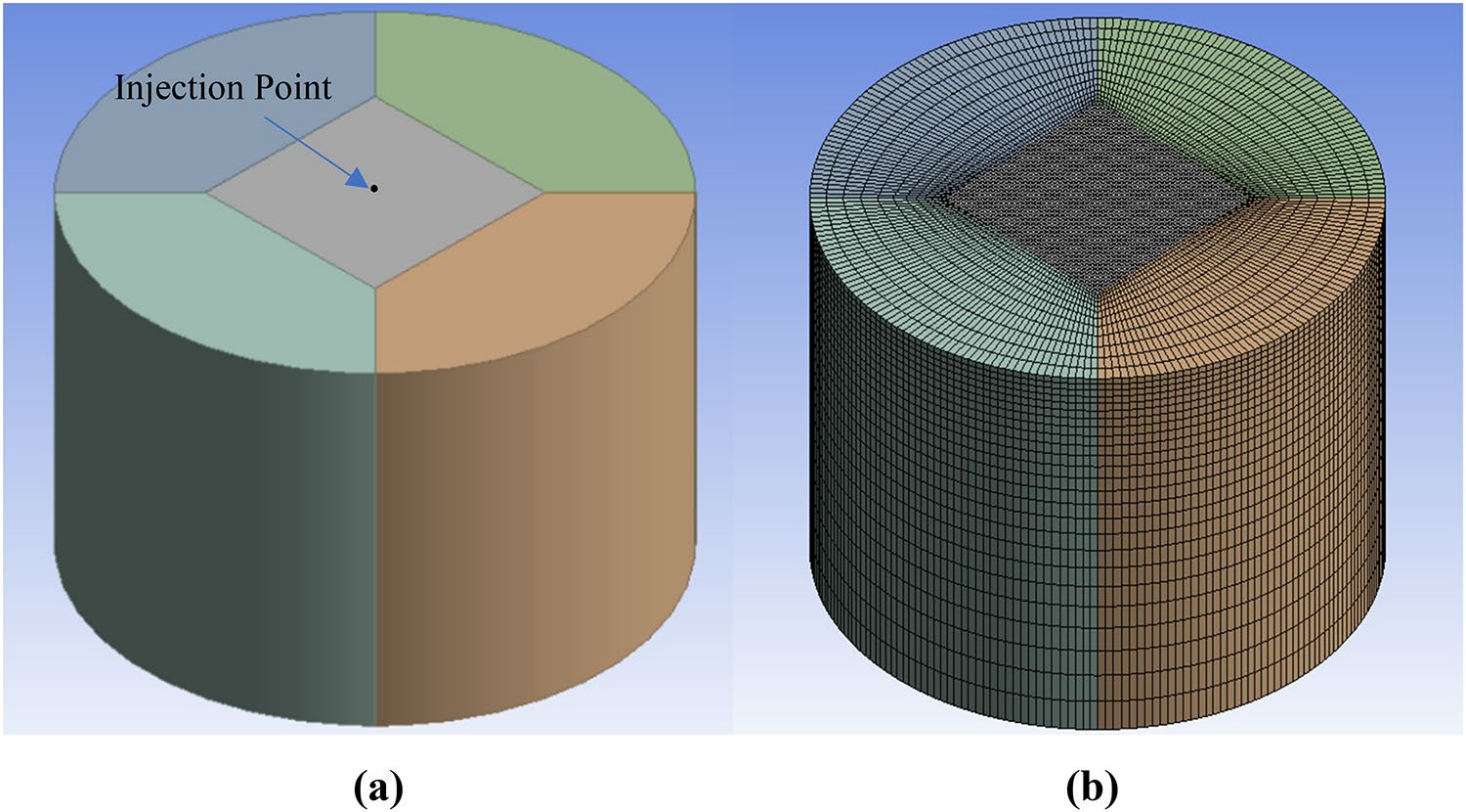
(**a**) Geometry and (**b**) Mesh of constant volume chamber.

ANSYS Fluent 2021-R1 is used to study the spray characteristics of fuel blends. A cylindrical domain with a diameter of 160 mm and length of 110 mm is designed to model the constant volume chamber. Further, a hexahedral mesh is generated using the ANSYS Meshing tool as it is having higher accuracy and efficiency^45^. A mesh of 149,625 elements and 158,600 nodes is finally selected to conduct the analysis after mesh independence is performed with different meshes. Figure 2a shows the Geometry and Fig. 2b Mesh of constant volume chamber.

The two-equation Standard k-ε model is used to model the turbulence. This model is based on model transport equations for the turbulent kinetic energy (k) and its dissipation rate (ε). This model is often influenced by its capacity to maintain a balance between computational efficiency and accuracy. It effectively captures turbulence characteristics in regions characterized by significant velocity gradients, making it suitable for spray analysis where an accurate representation of breakup and dispersion is crucial^46–48^.

The two-way turbulence method is used as it considers the interaction between the discrete and continuous phases. The dispersion of particles due to the turbulence in the fluid phase is predicted with the discrete random walk model. A pressure swirl atomizer is used to inject the fuel into the chamber, as it can produce fine and stable spray even at higher pressures and at lower injection rates. The various injection parameters used in the present work are presented in Table 2. Nozzle diameter and chamber pressure are taken from the literature published earlier^37,49,50^. The spray tip penetration (penetration length) can be measured using DPM report definitions available in ANSYS Fluent. With the data obtained from report definitions, the penetration length vs time plots are drawn.

**Table 2:** Injection parameters used for simulations.

Point properties	Value
Injection pressure	30 MPa
Fuel temperature	290 K
Chamber pressure	2 MPa, 2.5 MPa
Ambient temperature	290 K
Nozzle diameter	0.126 mm, 0.15 mm, 0.2 mm

For pressure–velocity coupling, second-order discretization and coupled schemes are used. The solution convergence is assumed when all residuals become smaller than 1E-6. The properties of the blends used for the study of spray analysis are presented in Table 3. The specific gravity and kinematic viscosity of the fuels are tested as per standards of ASTM D1298 and ASTM D445 respectively^51,52^. To conduct the analysis, different cases are considered for the simulation by varying the diameter of the injector nozzle and varying the chamber pressure by keeping the injection pressure and injection temperature constant for the different fuel blends. The different test cases considered for simulation are presented in Table 4.

**Table 3:** Properties of fuel blends used for simulations.

Fuel blends	Density (kg/m^3^)	Kinematic viscosity (mm^2^/s)	Surface Tension (N/m)	Specific Heat (J/kg-k)
D100	840	2.4	0.026	2090
D80B20	848	2.611	0.0272	2060
D80B10E10	839	2.239	0.0255	2113
D80B10HE10	841	2.303	0.0258	2121

**Table 4:** Test cases used for simulations.

Test case	Case 1	Case 2	Case 3	Case 4
Injection pressure	30 MPa	30 MPa	30 MPa	30 MPa
Injection temperature	290 k	290 k	290 k	290 k
Chamber pressure	2 MPa	2 MPa	2 MPa	2.5 MPa
Nozzle diameter	0.126 mm	0.15 mm	0.2 mm	0.126 mm

## Results and discussion

### Model validation

**Figure 3:**
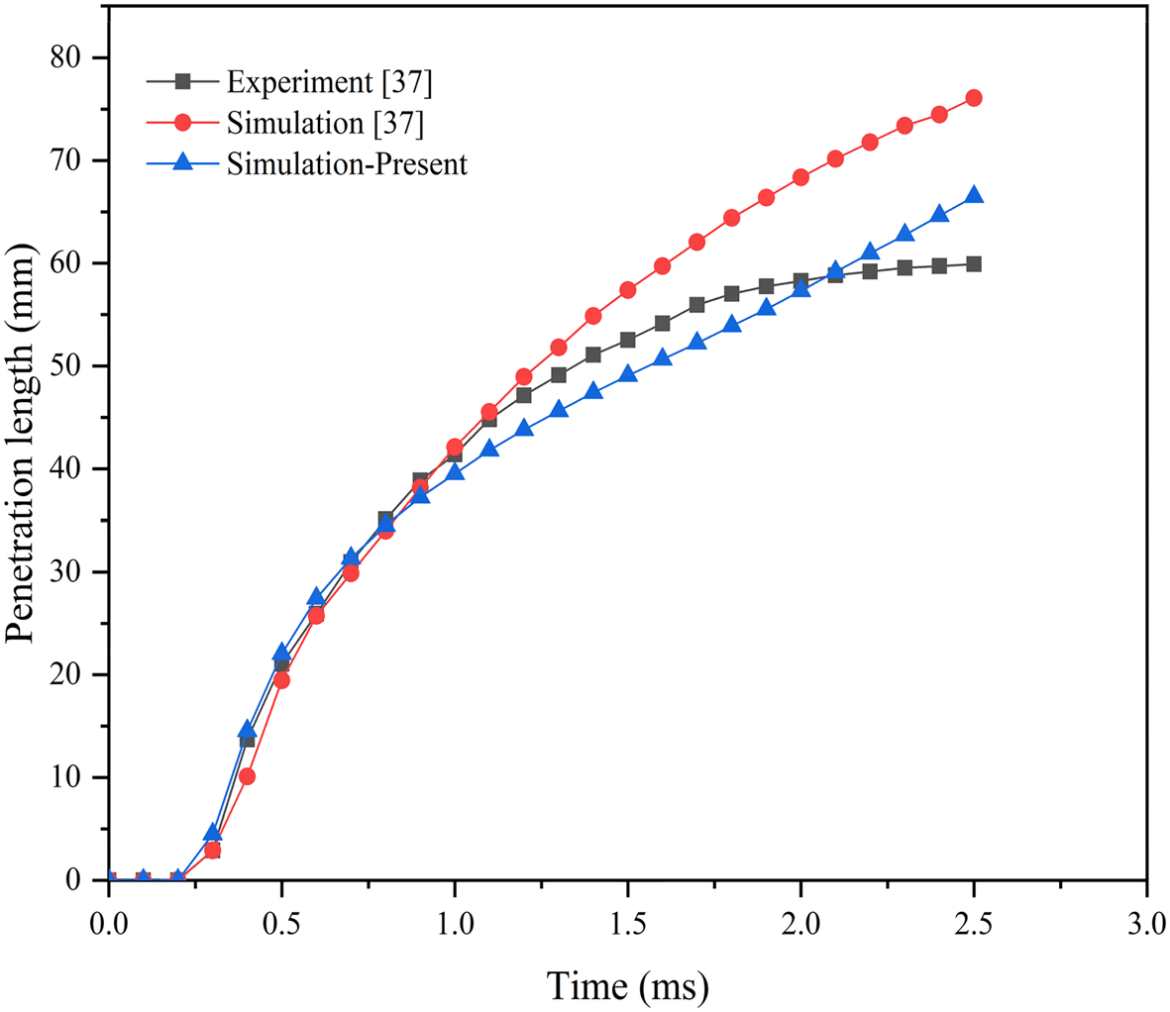
Penetration length from experimental and simulation results.

It is essential to evaluate the model's accuracy and reliability before simulating it and using the specified settings. For validation purposes, the available results from Park et al.^37^ are selected. For this, the simulations are performed by adopting similar geometrical and operating parameters as considered by Park et al.^37^. Further, the simulated results of spray penetration diesel fuel are used for the comparison. Figure 3 represents the comparison of simulated and experiment results of Park et al.^37^ with diesel fuel. It can be seen from Fig. 3 that that the simulated penetration length follows a similar trend as the experimental penetration length. The average error for the spray penetration length between the experiment results by Park et al.^37^ and current simulations is 1.35%.

### Mesh Independence

**Figure 4:**
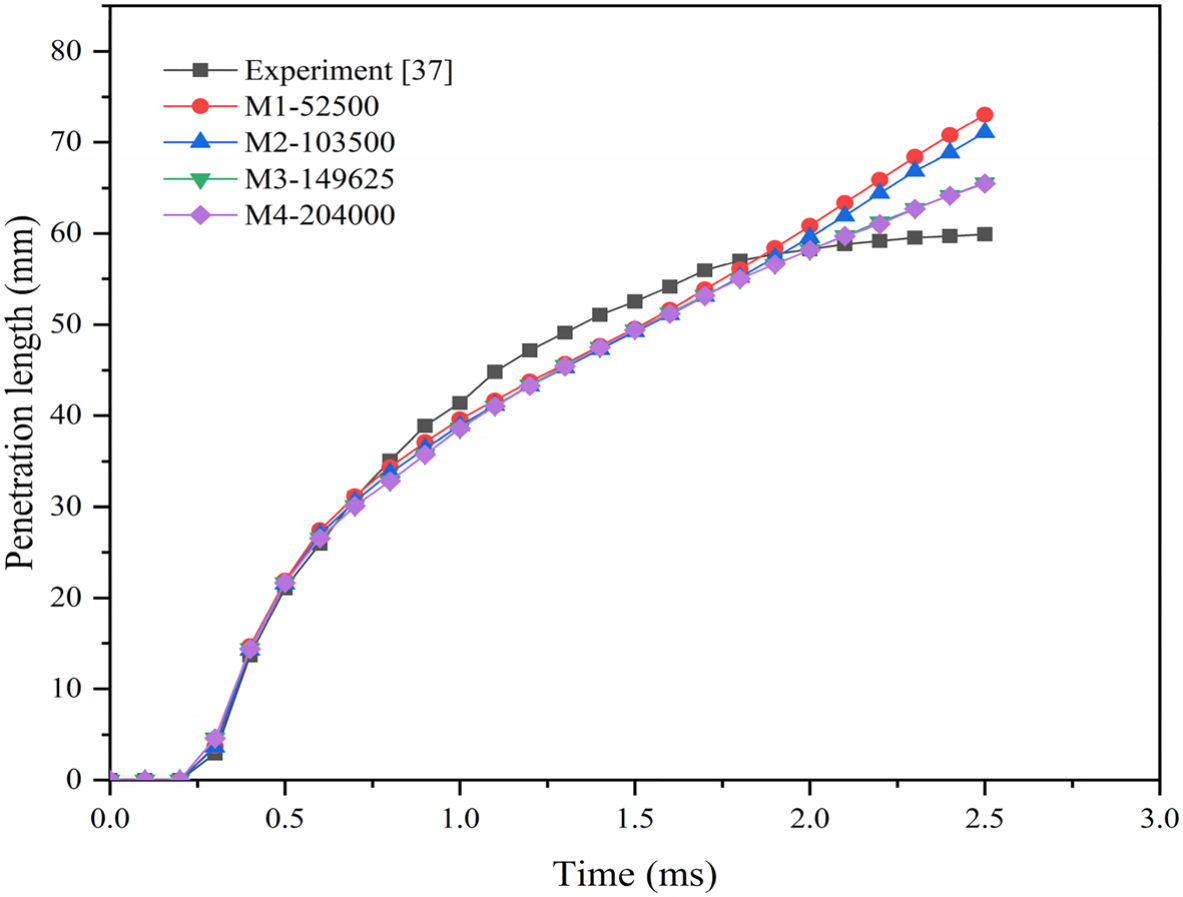
Penetration length for different mesh sizes.

Figure 4 shows the penetration lengths for different grid sizes used for grid independence study. The grid sizes are selected based on varying the edge sizing parameters of the domain. Four different grid sizes are investigated for penetration lengths by taking the experimental penetration length by Park et al.^37^ as the reference. The different grid sizes chosen are M1 with 52,500 elements, M2 with 103,500 elements, M3 with 149,625 and M4 with 204,000 elements. As the grid size is increasing, penetration lengths tend towards the experimental values. The average error percentage between the experimental and M3, M4 is 0.907% and 0.81% respectively. By increasing the grid size from M3 to M4, the variation in penetration lengths is not very high. So, the grid M3 is chosen for computational study of the spray characteristics.

### Effect of fuel blends on penetration length

**Figure 5:**
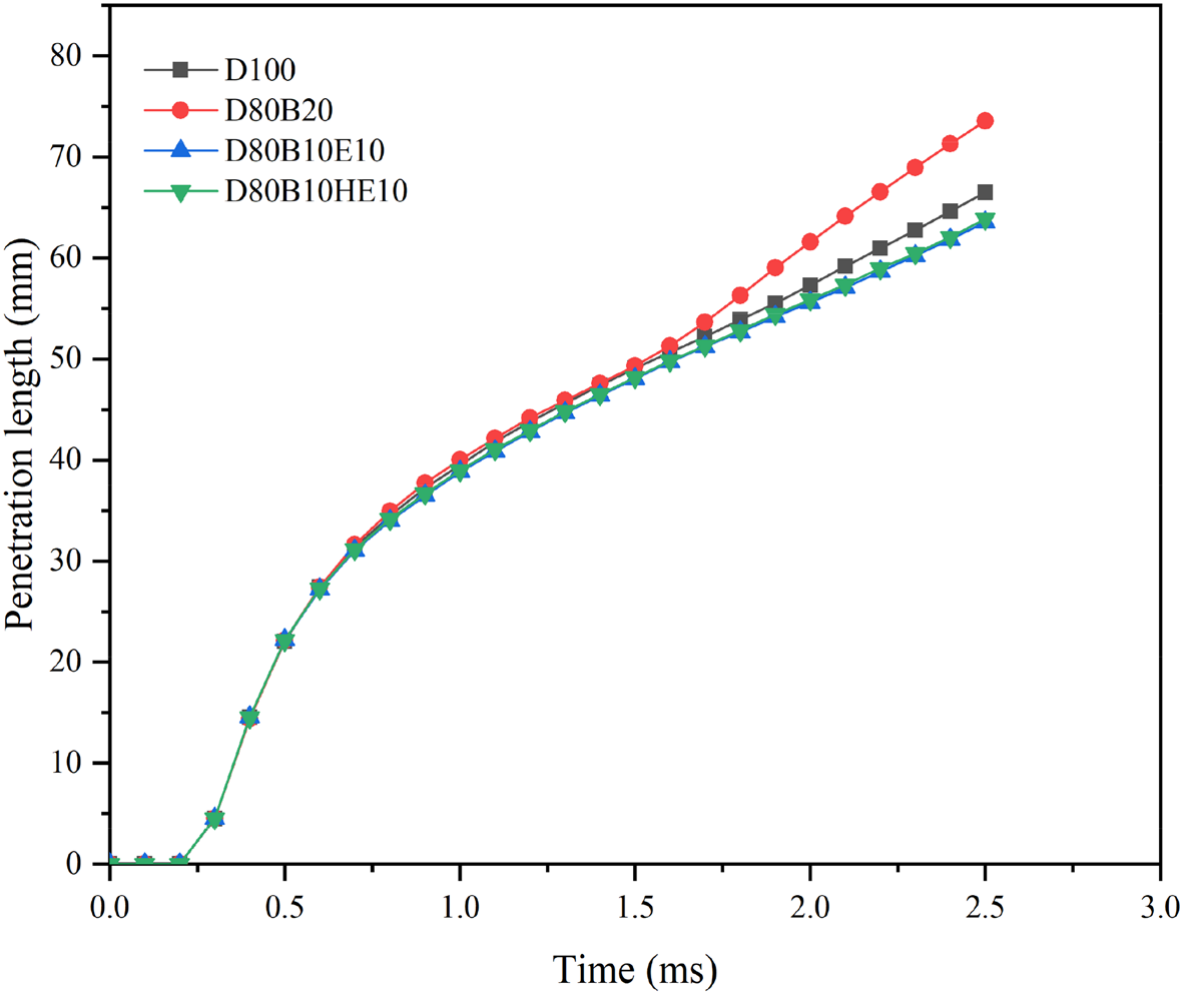
Penetration length of various fuel blends at 2 MPa ambient pressure and 0.126 mm nozzle diameter.

To understand the effect of fuel blends on spray penetration length, the analysis is performed by using the fuel blends mentioned in Table 1. Figure 5 shows the variation in spray penetration length for different fuel blends. It shows that the penetration length varies for different fuel blends. The diesel–biodiesel blend has having higher penetration length compared to pure diesel and diesel and ethanol blends. This may be due to the higher density and viscosity of the diesel–biodiesel fuel blend. The increase in density increases the mass flow rate, which in turn imparts higher momentum to the spray jet. Thus, the force applied to the surrounding stationary air increases, which may increase the penetration of spray into the chamber. Further, it is observed that the rate of increase in penetration length is initially more and then decreases. This may be due to the high kinetic energy of the fuel droplets initially, which is later decreased by the effect of resistance offered by the surrounding air^53–55^. Increase in viscosity of fuel increases the penetration length. This is may be due to the reduction in the atomization capacity of the fuel blend with the increase in the viscosity^56,57^.

### Effect of nozzle diameter on penetration length

**Figure 6:**
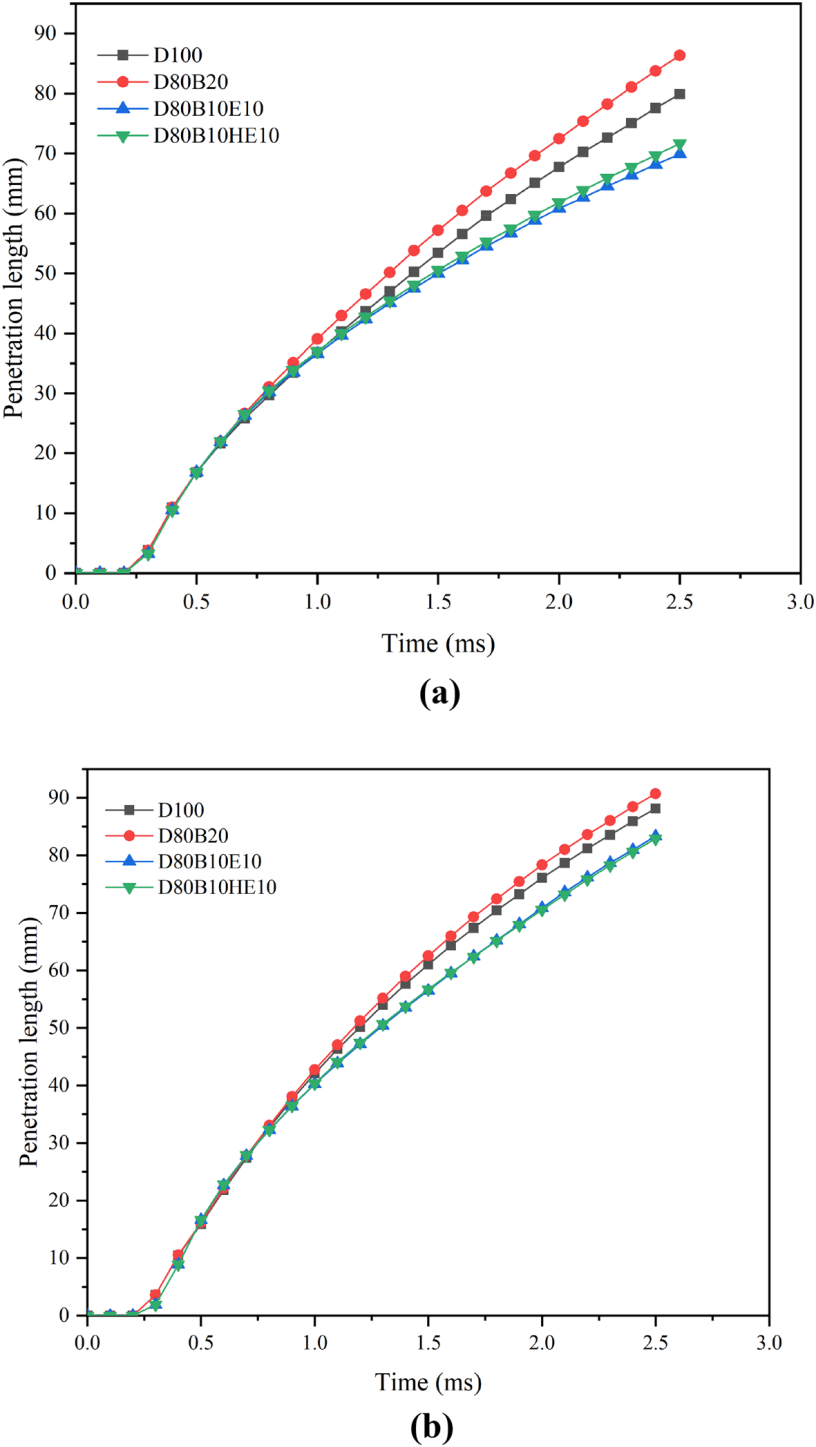
(**a**) Penetration length of various fuel blends at chamber pressure and nozzle diameter of 2 MPa and 0.15 mm. (**b**) Penetration length of various fuel blends at chamber pressure and nozzle diameter of 2 MPa and 0.2 mm.

**Figure 7:**
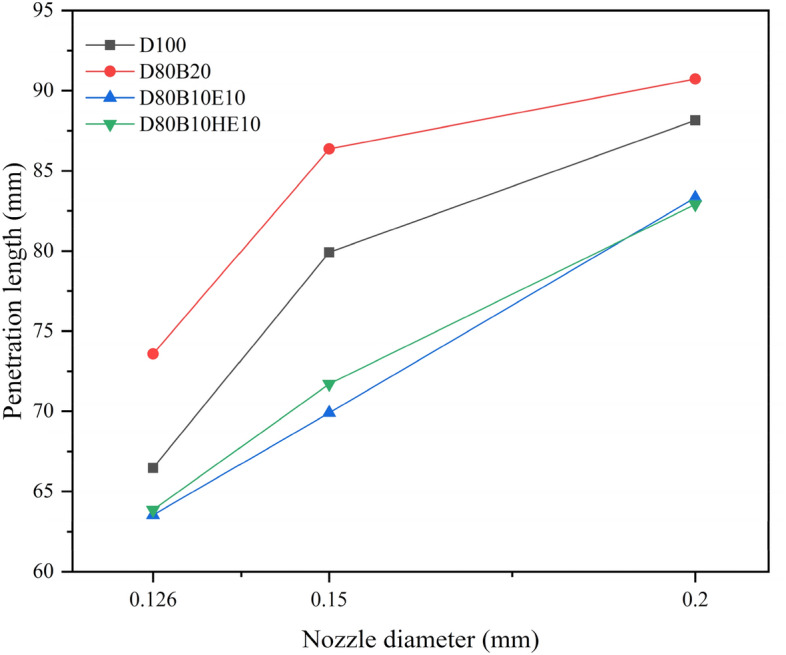
Comparison of Penetration length of various fuel blends at different nozzle diameters (after 2.5 ms).

To understand the effect of nozzle diameter on the spray penetration, the analysis is performed with three different nozzle diameters of 0.126 mm, 0.15 mm, and 0.2 mm. Figure 6a,b presents the variation in the spray penetration length with time for different nozzle diameters. It is observed that by increasing the injector nozzle diameter, the penetration length of the fuel blends is increasing. Similar results are also noticed by^32,58,59^. This may be due to the decrease in the velocity of injection while keeping the flow rate constant. The lower velocity of the spray improves the spray breakup and mixing. Large droplets are formed during the primary breakup, and these larger droplets are dispersed into finer droplets during the secondary breakup, which increases the spray penetration into the chamber. The geometry of the spray cone and the trajectory of fuel droplets are influenced by the nozzle diameter. A larger nozzle diameter may result in a wider spray cone, contributing to increased penetration. A comparison of penetration length with respect to diameter is presented in Fig. 7. It shows that by increasing the injector diameter, the difference in the penetration length of blends decreases.

### Effect of chamber pressure on penetration length

**Figure 8:**
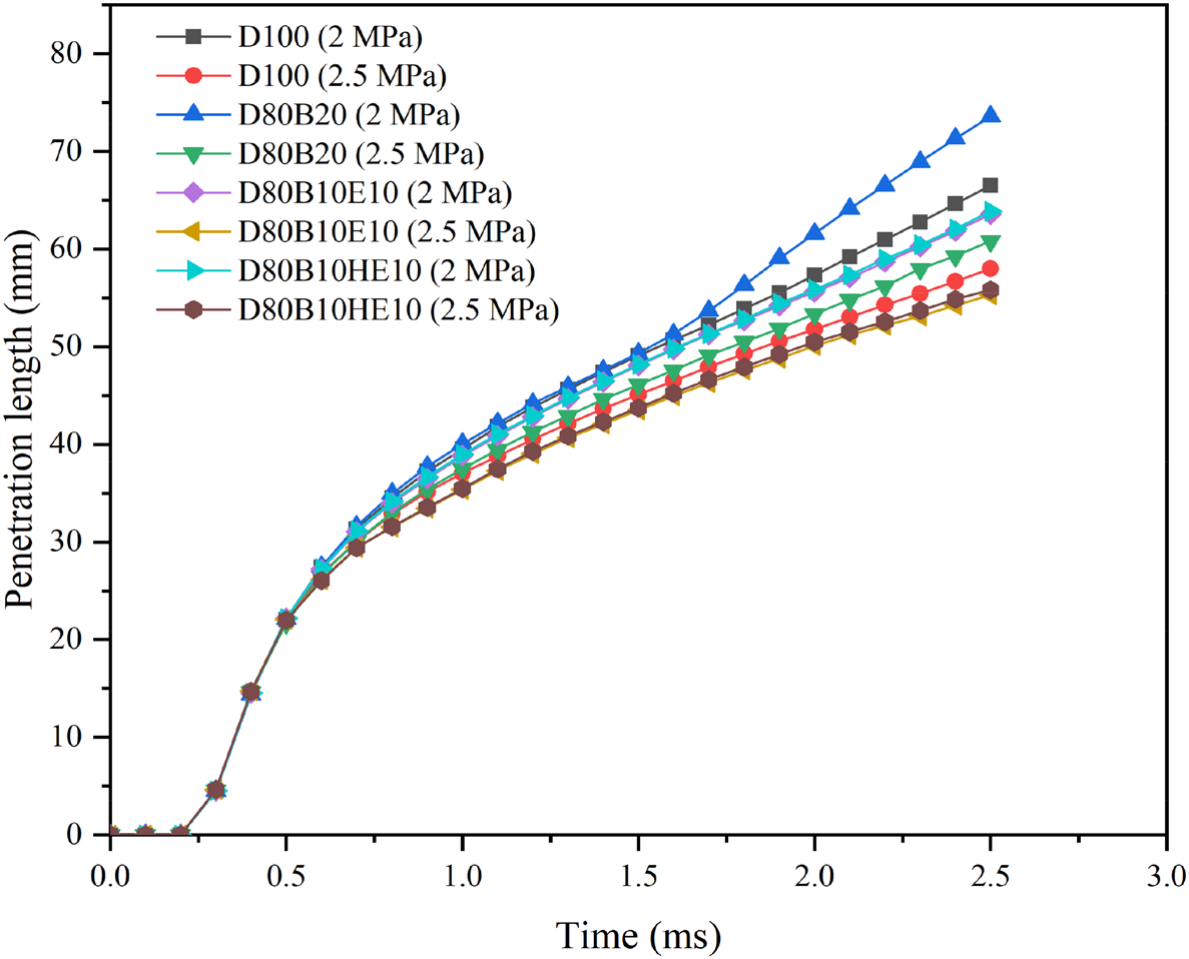
Comparison of Penetration length of various fuel blends.

The effect of chamber pressure on spray penetration length is also analyzed. For this, the simulations are conducted at a chamber pressure of 2.5 MPa and 2 MPa. Figure 8 shows the spray penetration length of different fuel blends used with 2 MPa and 2.5 MPa chamber pressure and 0.126 mm injector diameter of nozzle. For all the test cases simulated, diesel–biodiesel (D80B20) shows higher penetration lengths compared with the other blends. The increase in chamber pressure decreases the penetration length for all the blends. A similar kind of result was reported by^27,55,60^. This may be due to the increase in the chamber pressure increases the density of air in the chamber. As the density increases, resistance to the flow of fuel droplets increases, which could reduce the penetration length of the fuel.

### Spray velocity distribution

**Figure 9:**
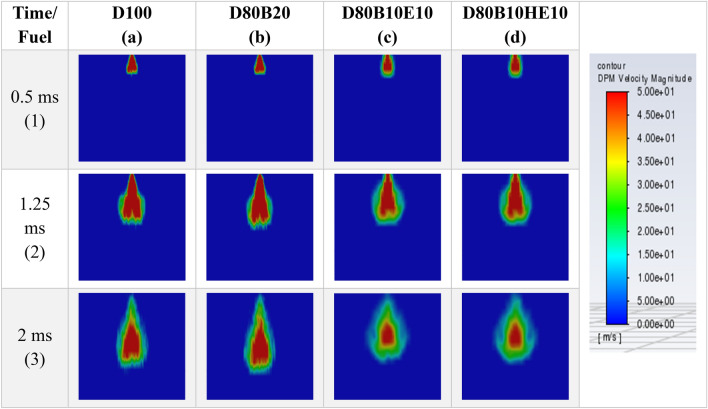
Fuel Velocity magnitude contours for different blends at 2 MPa chamber pressure and 0.15 mm nozzle diameter.

**Figure 10:**
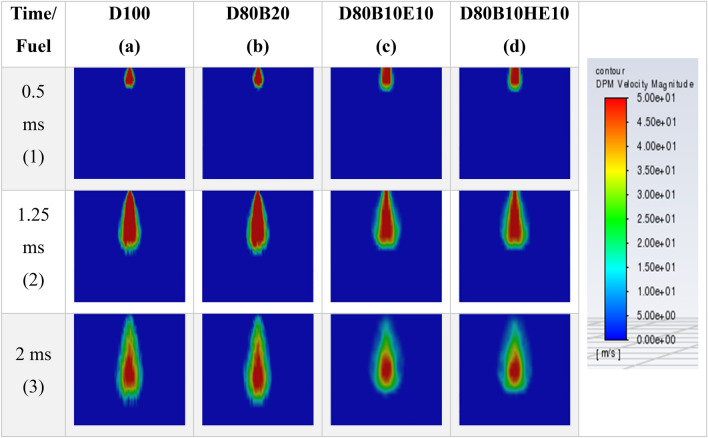
Fuel velocity magnitude contours for different blends at 2 MPa chamber pressure and 0.2 mm nozzle diameter.

To understand the spray velocity variation with flow time, contours of spray velocity magnitude are analyzed. Figures 9 and 10 shows the fuel spray velocity magnitude contours concerning time for 0.15 mm and 2 mm diameter nozzle, respectively. A high-velocity zone at the center of the spray is observed, while the outer region has low velocity. This may be due to the outer region experiencing resistive forces from the gas entrained in the chamber. Thus, the center zone moves with higher velocity compared to the outer region.

Further, as the spray advances into the chamber, it develops in axial direction as well as in radial direction. All the blends exhibit almost the same penetration length up to 0.5 ms just after the fuel injection. After that, there is a change in the penetration length of the spray depending on the fuel blend. After the injection process is complete, the spray moves in a similar path, but the velocity of the fuel particle is decreased. The velocity of fuel particles is less for blends of diesel-ethanol and diesel-hydrous ethanol.

## Conclusion

A numerical study was carried out to study the spray characteristics of diesel, waste cooking oil biodiesel, and hydrous ethanol blends under non-evaporating conditions. The DPM model, along with other sub-models, is used for the simulation study of spray. The effects of various blends of diesel fuel with the change in chamber pressure and nozzle diameter are investigated. The penetration length is affected by the fuel properties like density and viscosity. D80B20 blend has a higher penetration length of about 10.695% and 15.805% than the D100 and D80B10HE10 blends, respectively, because of its high density. With the increase in nozzle diameter, the penetration length of diesel fuel is increased by 20% and 32% for 0.15 mm and 0.2 mm, respectively. It can also be noted that with an increase in nozzle diameter from 0.126 mm to 0.2 mm, the difference in penetration length of fuel blends decreases from 10.695% and 15.805% to 2.935% and 8.895% for D100 and D80B10HE10 respectively. With the change in chamber pressure from 2 MPa to 2.5 MPa, the penetration length for diesel is decreased by 14.62%. With an increase in pressure, chamber air density increases, which decreases the penetration length. Similar to the change in nozzle diameter, with an increase in chamber pressure, the difference in penetration length is decreasing. From the different plots of penetration length, it can be observed that the penetration length of the D80B10E10 and D80B10HE10 blend is lesser than the pure diesel, and it affects the combustion and emissions of the engine. From the different plots, it can be observed that there is not much variation in penetration lengths between D80B10E10 and D80B10HE10 because the properties of the fuel blend are very close to each other. The use of hydrous ethanol over pure ethanol is cost-saving and energy-saving. So, hydrous ethanol with the appropriate amount of water content can be blended with diesel and biodiesel. The study of spray characteristics of biofuels will help to optimize the injection operating parameters for use in internal combustion engines.

## Future scope

Numerical analysis of spray can be extended to evaporative cases and also fuel blends with higher hydrous ethanol content can also be performed. Other macroscopic properties of spray can also be investigated numerically. Further, the combustion modeling can be performed with the different fuel blends using different combustion models available in the CFD packages.

## Data Availability

The datasets used and/or analysed during the current study available from the corresponding author on reasonable request.
